# Aesthetic Motivation Impacts Judgments of Others’ Prosociality and Mental Life

**DOI:** 10.1162/opmi_a_00113

**Published:** 2023-12-08

**Authors:** Tanushree Agrawal, Adena Schachner

**Affiliations:** Department of Psychology, University of California San Diego

**Keywords:** interpersonal judgments, prosocial behavior, beauty, aesthetics, emotionality

## Abstract

The ability to infer others’ prosocial vs. antisocial behavioral tendencies from minimal information is core to social reasoning. Aesthetic motivation (the value or appreciation of aesthetic beauty) is linked with prosocial tendencies, raising the question of whether this factor is used in interpersonal reasoning and in the attribution of mental capacities. We propose and test a model of this reasoning, predicting that evidence of others’ aesthetic motivations should impact judgments of others’ prosocial (and antisocial) tendencies by signaling a heightened capacity for emotional experience. In a series of four pre-registered experiments (total *N* = 1440), participants saw pairs of characters (as photos/vignettes), and judged which in each pair showed more of a mental capacity of interest. Distractor items prevented participants from guessing the hypothesis. For one critical pair of characters, both characters performed the same activity (music listening, painting, cooking, exercising, being in nature, doing math), but one was motivated by the activities’ aesthetic value, and the other by its functional value. Across all activities, participants robustly chose aesthetically-motivated characters as more likely to behave compassionately (Exp. 1; 3), less likely to behave selfishly/manipulatively (Exp. 1; 3), and as more emotionally sensitive, but not more intelligent (Exp. 2; 3; 4). Emotional sensitivity best predicted compassionate behavior judgements (Exp. 3). Aesthetically-motivated characters were not reliably chosen as more helpful; intelligence best predicted helpfulness judgements (Exp. 4). Evidence of aesthetic motivation conveys important social information about others, impacting fundamental interpersonal judgments about others’ mental life and social behavior.

## INTRODUCTION

When encountering a new person, one of the first things people judge is how likely they are to behave prosocially, i.e., in a beneficial vs. harmful way towards others (Fiske et al., 2007; Wojciszke, 2005). Judgments about social and moral qualities such as honesty, generosity, helpfulness, trustworthiness, or compassion (versus selfishness, deceptiveness, or manipulativeness) are important because they are likely to directly impact the well-being of the perceiver (Winter & Uleman, 1984; Wojciszke, 2005; Wojciszke et al., 1998). These qualities notably all relate to the extent to which one values and promotes others’ goals and desires, an inference which is at the core of social reasoning (Jara-Ettinger et al., 2016; Pesowski et al., 2023; Pfattheicher et al., 2022; Powell, 2021).

In particular, the ability to reason about others’ likelihood of prosocial vs. antisocial behavior from brief snippets of information appears to be a fundamental part of social reasoning. People readily use brief observations of prosocial or antisocial behavior to infer a more general trait or tendency (Asch, 1946; Borkenau et al., 2004; Kenny et al., 1992; Winter & Uleman, 1984), and to make predictions about a person’s future prosocial or antisocial actions (Back et al., 2010; McCrae & Costa, 1995). People seek out information about moral traits more actively, and weigh this information more heavily, than anything else when characterizing new people as friends or foes (Fiske et al., 2007). The foundations of this reasoning emerge early in life: Within 6 months of age, infants use brief observations of others’ helpful or harmful behavior to decide who to approach or avoid, and to form expectations about who others will approach or avoid (Hamlin & Wynn, 2011; Hamlin et al., 2007; Kuhlmeier et al. 2003; Powell & Spelke, 2018).

People also use more subtle aspects of appearance and behavior to draw conclusions about others’ prosocial or antisocial tendencies. Non-verbal behaviors like gestures, body postures, and facial expressions, as well as attributes of clothing and other possessions, lead to inferences about others’ personality and prosociality from even a 30-second glimpse (Allport, 1937; Ambady & Rosenthal, 1992; Babad et al., 1989; Burroughs et al., 1991; Streeter et al., 1977). Inferred membership in social categories—based on cues to race, gender, and social group—also activates stereotypes about those particular social groups and their members (Cosmides et al., 2003; Hugenberg & Sacco, 2008; Macrae & Bodenhausen, 2001), shaping social and mental trait inferences in potentially inaccurate ways, including those about expected prosociality/antisociality (Ashmore & Del Boca, 1981; Petsko & Bodenhausen, 2020; Rubinstein et al., 2018, 2023). Lastly, people readily (and often inaccurately) attribute prosocial traits like agreeableness and antisocial traits like cruelty to unfamiliar others based on facial features alone (Berry & Finch Wero, 1993; Lin et al., 2021).

Whether accurate or not, inferences drawn about others from these subtle features have broad consequences for social interaction and real-world social behavior. Adults and even children use these cues to decide who to be friends with (Afshordi & Liberman, 2021; Powell, 2022), who to avoid (Hamlin & Wynn, 2011; Hamlin et al., 2007; Kuhlmeier et al. 2003; Powell & Spelke, 2018), and even who it is acceptable to harm (Gray et al., 2007; Goodwin, 2015; Liberman et al., 2017; Rhodes & Chalik, 2013). Thus, it is important to understand the ways in which surprising or seemingly irrelevant features of behavior impact fundamental judgements of others’ social and moral qualities.

### Aesthetic Appreciation May Impact Social Judgements

Here we test the idea that social and moral interpersonal judgements are shaped by a factor not previously considered: Others’ aesthetic behaviors, i.e., engagement in art forms like music or painting; and aesthetic motivations, i.e., the value or appreciation of aesthetic beauty, including in activities that are not traditionally considered art. Aesthetic behaviors, like engagement with visual art or music, are common to everyday life in cultures around the world (Brown, 2004; Mehr et al., 2019; Savage et al., 2015), and are not recent cultural inventions but ancient, fundamental components of human behavior (e.g., Conard et al., 2009; Fazenda et al., 2017; Jacoby et al., 2021; Sugiyama, 1996; Tooby & Cosmides, 2001). Outside the context of conventional aesthetic activities, the appreciation of beauty is central to human experience, motivating behavior in a broad range of contexts from love and attraction to the pursuit of scientific knowledge (Diessner, 2019). The appreciation of aesthetic beauty is linked with powerful feelings of hope and spirituality (Diessner et al., 2006, 2013), and aesthetic preferences have been argued to be adaptive and shaped by natural selection (Dutton, 2003, 2009).

In modern-day Western cultures, there are marked individual differences in the extent to which people engage in aesthetic activities, and in the extent to which people’s behaviors are motivated by an appreciation of aesthetics and beauty (Diessner et al., 2008; Fayn et al., 2015; Schlotz et al., 2021). As a result, if individual differences in aesthetic activities and motivations impact fundamental social inferences like others’ prosociality, this factor would have broad consequences for social perception.

One compelling reason to think that observing others’ capacity for aesthetic engagement would impact judgments of their prosociality is that this judgment may be grounded in reality: Aesthetic engagement appears to be correlated with prosocial tendencies. Across large, nationally-representative samples, arts engagement has been found to positively correlate with prosocial traits (helping attitudes and dispositional empathy) and prosocial behaviors (charitable donations, volunteering, and real-world helping behaviors)—even after accounting for sociodemographic variables like age, gender, education, religious attendance, income levels, political orientation, and overall physical health (Kou et al., 2020; Van de Vyver & Abrams, 2018). This pattern holds across a wide variety of aesthetic activities, including the consumption and creation of visual art (e.g., visits to art museums and galleries, purchases of artworks, and art-making activities like painting, pottery, woodworking, and quilting), performance art (e.g., participation or attendance at music, dance, or theatrical performances), and literature (e.g., reading for pleasure, and creative writing; Kou et al., 2020). Previous domain-specific work has found similar links between prosocial behavior and engagement in visual arts, music, dance, theater, and literature, each considered individually (for a comprehensive review, see Konrath & Kisida, 2021; see Tay et al., 2018 for a related theoretical framework).

The capacity for aesthetic *appreciation* also appears to predict prosocial tendencies. The capacity for aesthetic appreciation—interest in beauty, and tendency to create or seek out art, music, poetry, or related stimuli—is a primary component of a personality trait known as ‘Openness to Experience’ (Christensen et al., 2019; Fayn et al., 2015; McCrae & Costa, 1997). Openness to experience is a reliable predictor of people’s artistic creativity (Feist, 1998; Silvia et al., 2009), their tendency towards arts-related professions (Barrick et al., 2003), and individual differences in aesthetic appreciation (Vessel & Rubin, 2010).

Crucially, people with higher levels of openness, and thus, higher capacities for aesthetic appreciation, have been found to be more prosocial: They exhibit more cooperative behavior (Klein et al., 2019), more agreeable behavior during interpersonal interactions (Fayombo, 2010; Shi et al., 2018), more complex moral reasoning skills (Dollinger & LaMartina, 1998), and lower levels of racial prejudice (Flynn, 2005), homophobia (Cullen et al., 2002), and authoritarianism (Perry & Sibley, 2012). These effects remain after accounting for demographic factors and individual differences in intelligence (e.g., Dollinger & LaMartina, 1998). In another series of large-scale studies, individual differences in appreciating and engaging with beauty specifically (whether artistic, natural, or moral beauty) were correlated with moral attributes including care, benevolence, universalism, agreeableness, and empathy (Diessner et al., 2013).

These findings are in line with philosophical theories that those who enjoy aesthetic endeavors have a higher moral character (Bicknell, 2001; Cox & Levine, 2016; Kivy, 2009). Some philosophers have further argued that aesthetic engagement itself plays a causal role in enhancing people’s interpersonal empathy, particularly in the domains of music (Ansani et al., 2019), art (Pizarro et al., 2006), and dance (Scruton, 1999).

This prior research suggests that people who appreciate aesthetic activities also tend to behave more prosocially. However, these past studies do not provide insight into how this feature may impact the social perception of others. Do people intuitively link aesthetic appreciation with prosocial behavior when predicting the prosocial and antisocial tendencies of new people? If people take aesthetic appreciation into account, they should infer that those who are more motivated by aesthetics are also more likely to behave prosocially.

### A Causal Model: How Aesthetic Engagement May Impact Judgments of Prosociality

**Figure 1.**
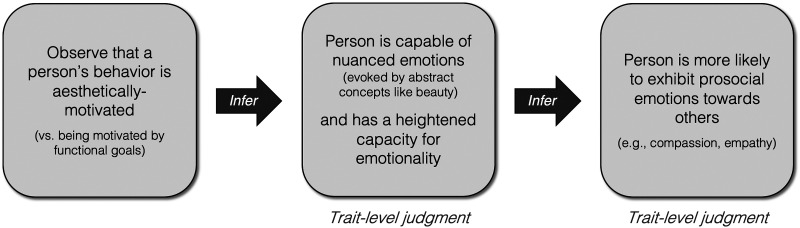
Hypothesized Causal Model. Observing others engaging in an activity for aesthetic reasons (motivated by an appreciation of beauty) may indirectly impact judgments about their prosociality by signaling that they have a heightened trait-level capacity for emotionality.

How might others’ aesthetic engagement impact judgments of prosociality? We hypothesize that inferences about emotional sensitivity mediate the link between others’ aesthetic engagement and inferred prosocial dispositions. In particular, our hypothesized causal model (see Figure 1) predicts that observers will make two key inferences. Firstly, we predict that observers will infer that individuals who appreciate aesthetic beauty possess a greater capacity for emotionality, or emotional sensitivity. Second, we predict that observers will infer that those with greater emotionality are more likely to behave prosocially, and less likely to behave antisocially.

The hypothesis that emotionality is an important mediator between aesthetics and prosociality is deeply rooted: Eighteenth-century philosophers like Hume, Shaftesbury, and Hutcheson believed that the ability to appreciate aesthetics and the ability to make moral, prosocial decisions were both driven by a common underlying capacity for emotionality (Dillon, 2004; Townsend, 2001). Sentimentalist philosophy hinges on the idea that the intrinsic human capacity for feeling emotions drives most aspects of behavior, including both morality and aesthetic engagement; and thus that these two factors are connected by a common underlying driver of emotionality (Heinzelmann et al., 2020; Hume, 1910). This theory is supported by empirical work showing links between aesthetic engagement and emotionality (e.g., individuals high in trait openness, who appreciate aesthetic beauty, also tend to show greater levels of emotional arousal, Fayn et al., 2015), as well as between moral standing and emotionality (e.g., characters who are judged as having a greater capacity for emotional experience tend to also be judged as being more morally wrong to harm, Gray et al., 2007).

We hypothesize that this type of sentimentalist intuition may also drive people’s third-person judgments about others’ aesthetic behaviors, such that observing an individual engaging in aesthetic behavior will impact judgments about their social and moral traits by signaling their underlying capacity for emotionality. Recent work on musical engagement is consistent with this idea: In controlled experiments, people judged musical characters more emotionally sensitive, and also more wrong to harm, than matched neutral or explicitly non-musical characters (Agrawal et al., 2023).

We hypothesize that what will impact these inferences most powerfully is not simply observing a particular behavior (like engaging with music), but observing evidence of aesthetic *motivation*: finding intrinsic value in the activity itself. Aristotle distinguished this category of action as *atelic* (vs. telic). While much of human behavior involves *telic* activities that are motivated by functional goals, like reaching an object or other external outcome (Kruglanski & Szumowska, 2020; Woodward, 1998), *atelic* activities are done for their own sake, rather than as the means to an end (Pigliucci, 2019; Schachner & Carey, 2013; Schmid, 2011). Aesthetic appreciation, or what we here term aesthetic motivation, involves pleasure that is atelic—free from external interests (Kant, 1987), “art for art’s sake” (Haskins, 1990).

People often engage in aesthetic activities like listening to music or attending arts performances for atelic reasons, simply because they enjoy them or find beauty in them (e.g., Swanson et al., 2008). However, people can engage in stereotypically aesthetic activities for telic reasons. For example, imagine a painter motivated not by love of art, but by a large cash prize in a competition. Similarly, factory workers who mass-produce artistic objects are seen as having extrinsic, profit-based motivations, while artists who develop unique pieces are seen as driven by their love for the art itself (Judge et al., 2020; Newman & Bloom, 2012). People can also engage in stereotypically functional activities for aesthetic, atelic reasons. For example, stereotypically functional activities can become atelic when one is fully immersed in the activity, in a state of flow (Csikszentmihalyi, 1990, 2000). People can also infer aesthetic motivations for activities not traditionally considered to be art, like when an individual is deeply moved by a beautiful sunset or landscape (Jorgensen, 2011; Moore, 2007), excited about the gastronomic complexity of food (Plakias, 2021), or sees elegance in numbers and mathematical proofs (Cellucci, 2015).

Individuals vary in their propensity to engage in activities for aesthetic, atelic reasons, vs. to be driven by telic goals (Baumann, 2021; Nakamura & Csikszentmihalyi, 2014). In philosophy, this tendency to value things for their own sake has been theorized to predict an individual’s own moral aptitude (Jaworska, 1999; Watson, 2013). We hypothesize that this factor may play a role in interpersonal perception as well. Choosing to spend valuable time or money on an activity for no other reason than finding it intrinsically beautiful or enjoyable may signal that the person is motivated by emotional experience, and may therefore have greater emotionality in general. In contrast, choosing to engage in an activity for a functional reason does not provide evidence that the person experiences strong emotions, as there is an alternative explanation for why they are engaging in the activity (i.e., to achieve the desired functional outcome).

If people reason about others’ aesthetic vs. functional motivations in this way, then they may make different judgments about the emotionality of an individual who engages in the very same activity for these two different reasons, judging the aesthetically-motivated person to be more emotionally sensitive. For example, a person who listens to music because they find aesthetic value in it should be judged more emotionally sensitive than an individual who listens to music in order to maintain focus and be productive at work (a functional, external outcome). Motivation rather than the activity itself should predict people’s judgments: People should infer heightened emotional sensitivity for those with aesthetic motivations even for activities that are not considered conventional art forms, such as cooking, hiking, or doing mathematical proofs.

Alternatively, it is also possible that observers will infer higher emotionality for any person who engages in conventional art forms. These stereotypically aesthetic activities typically involve a high degree of emotionality (Larsen & Sackris, 2020; Menninghaus et al., 2019), and many people are intuitively aware of this. For example, people believe that strong emotional responses may be evoked while listening to music (Juslin, 2013; Petrides et al., 2006; Stewart, 2007; Zentner et al., 2008), watching dance performances (Fitzpatrick & Longley, 2014), viewing art installations, (Konečni, 2015), or reading narrative fiction (Mar et al., 2011). In the specific domain of musicality, recent experimental evidence has also shown that people judge musical characters as more emotionally sensitive than matched neutral or explicitly non-musical characters (Agrawal et al., 2023). If people believe that any engagement in conventional art activities provides evidence of emotionality, then they should make different judgments about peoples’ emotionality on the basis of activity type (conventional art forms like music or painting; vs. activities not considered conventional art forms, like hiking, cooking, or math), and should make similar social judgments for the same activity regardless of the individuals’ aesthetic vs. functional motivations.

Lastly, the model makes the further prediction that inferences about emotionality will lead to the inference that individuals with aesthetic motivations are more prosocial than those with non-aesthetic, functional motivations. This prediction is motivated by the finding that judgements about others’ capacity for emotional experience strongly impact a broad range of social and moral judgements (Epley & Waytz, 2010; Gray et al., 2007; Knobe & Prinz, 2008; Robbins & Jack, 2006; Sytsma & Machery, 2012; Weisman et al., 2017). In addition, individuals who show emotional expressivity (particularly positive emotion) are intuitively judged as more altruistic or cooperative (Brown et al., 2003; Mehu et al., 2007). Thus, in the current context, emotional sensitivity may be interpreted as evidence of prosocial tendencies, linking aesthetic motivation with prosocial attributions.

### The Current Studies

Here, we empirically test whether others’ aesthetic motivations, or alternatively any engagement in conventional art activities regardless of motivation, leads to judgments of increased prosocial and decreased antisocial qualities. Further, we test whether this effect is mediated by the inference of heightened capacity for emotionality. To test this, we conducted a series of four pre-registered experiments. Our experimental design builds on established methods (Agrawal et al., 2023; Gray et al., 2007), and was chosen in order to create tightly controlled experimental conditions, while also minimizing demand or expectancy effects (Robson, 2011; Rosenthal & Rubin, 1978).

To minimize demand effects, participants were asked to make judgments not only about characters of interest (two characters who engaged in an activity, for aesthetic vs. non-aesthetic reasons), but also about a number of distractor characters (e.g., a sociable robot, a frog, a baby, etc., adapted from Gray et al., 2007). In the test phase of each experiment, participants saw all possible pairings of characters, including the numerous distractor characters, and on each trial selected the character from the pair that showed more of the mental capacity of interest (e.g., repeatedly choosing which of two characters was more emotionally sensitive). Only one of these many trials was our critical comparison between the matched aesthetic versus functional characters; all other trials involved one or more distractor characters.

This experimental method had two useful design features. One, it effectively hid the purpose of the study from participants, such that almost no participants guessed the hypotheses or guessed that the critical characters were of particular interest. Second, it allowed us to check our manipulation and probe the validity of the task as a measure of social judgments. We did this by examining participants’ judgments across the full range of characters, including the distractors, to check that participants made sensible judgments that were in line with those in previous work (e.g., judging a baby more emotionally sensitive than a robot; Agrawal et al., 2023; Gray et al., 2007).

In addition, this experimental design allowed us to create tightly controlled experimental conditions. All characters were represented by a photo and a short vignette, which could then be counterbalanced and matched on features other than the manipulation of interest. The two critical, matched characters were both adults of the same gender, described as engaging in the same activity (e.g., listening to music). One character was described as motivated by the aesthetic beauty of the activity, and the other as motivated by the usefulness of that activity in achieving some functional goal. Within each pair of critical characters, descriptions were tightly matched for length, style, and type of information provided. For example, neither vignette included any direct mention of social interaction or prosocial/antisocial behavior, and both vignettes involved enjoyment and finding value in the activity. Crucially however, the aesthetically-motivated character enjoyed the activity itself, while the functionally-motivated character enjoyed an external outcome of the activity.

Between subjects, we varied the activity that the critical pair of characters engaged in, including both formal artforms (music, painting) and also other activities that can be motivated by either aesthetics or non-aesthetic functions (cooking, exercising, being in nature, and doing math). This allowed us to test the alternative hypothesis that only engagement in a formal art form would lead to increased prosocial attributions. In addition, by varying content and including multiple activities, we were able to ensure that findings were not driven by unique features of any particular vignette, and test the extent to which findings generalized across the variety of distinct activities and vignette contents.

Using this method, we first tested whether people choose aesthetically-motivated characters as being more prosocial and less antisocial than matched functionally-motivated characters, asking whether aesthetically-motivated characters are judged more likely to behave compassionately, and less likely to behave selfishly/manipulatively (Exp. 1). We then tested *why* others’ aesthetic motivations impact judgments about their social behavior, probing the hypothesis that this effect is mediated by perceptions of others’ broader mental capacities, particularly their capacity for emotionality (vs., for example, their intelligence). We tested this hypothesis firstly by analyzing whether people choose aesthetically-motivated individuals as more emotionally-sensitive than matched functionally-motivated ones (Exp. 2), and then by assessing whether perceptions of heightened emotionality predict and explain judgments about prosocial behavior. This experiment also provided the opportunity to replicate all prior findings (Exp. 3).

Finally, we tested whether aesthetic appreciation impacts inferences about all or only some aspects of prosociality, by exploring another fundamental prosocial trait: helpfulness (Penner et al., 2005). If aesthetic appreciation impacts inferences about all aspects of prosociality, then aesthetically-motivated people should be seen as more helpful, in addition to being seen as more compassionate towards others and less selfish/manipulative. Alternatively, if these prosocial effects are mediated by judgments of enhanced emotionality, we may see a distinct pattern for helpfulness: Effective helping requires more than the motivation to help, but also the intelligence, skill, and ability to help effectively (Dovidio, 1984). Thus, if aesthetic appreciation does not increase intelligence judgments, then aesthetic appreciation may not increase inferences about helpfulness (Exp. 4), despite reliably impacting judgements about other kinds of prosocial and antisocial behavior.

Overall, the design of these experiments allows us to (1) test the impact of our manipulation of interest (aesthetic vs. functional motivations) in a controlled manner; (2) investigate people’s complex interpersonal judgments without demand effects; and (3) test a wide range of aesthetic activities and interpersonal judgments in an equivalent way, allowing for direct comparisons across results, and statistical tests of our hypotheses. All experiments received ethics approval from the Institutional Review Board at the university where the work was conducted (protocol 161173), and written consent was obtained from all participants. Stimuli, data, methods, and supplemental material for all experiments can be found on OSF (https://osf.io/gmey5/?view_only=afd333b8c1574d428134c9ed5f21658c). We preregistered all experiments, and preregistration documents are also included in the OSF repository.

## EXPERIMENT 1

In a first experiment, we test whether people judge aesthetically-motivated characters as more prosocial and less antisocial than matched functionally-motivated characters. In particular, we ask whether aesthetically-motivated characters are judged more likely to behave compassionately, and less likely to behave selfishly/manipulatively.

Compassion is a highly prosocial trait, associated with feelings of warmth, concern, and care for others (Strauss et al., 2016). Here, we focus on the act of *behaving* compassionately, whereby an individual acts to alleviate others’ suffering (Gilbert, 2019, 2020; Goetz et al., 2010; Jazaieri et al., 2018; Kanov et al., 2004; Strauss et al., 2016). Compassionate behavior involves acting prosocially towards others by treating them with warmth, care, and concern, and is lacking in those who exhibit antisocial behaviors like bullying (Gini et al., 2011; Leiberg et al., 2011; Preckel et al., 2018; Singer & Klimecki, 2014).

Contrastingly, selfish and manipulative behaviors are fundamentally antisocial, and are associated with antagonistic personality characteristics like Machiavellianism, narcissism, and psychopathy (Christie & Geis, 2013; Jakobwitz & Egan, 2006; Jones & Paulhus, 2014). Peoples’ scores on indices of selfishness and manipulativeness as personality traits strongly predict antisocial behaviors such as overt aggression (Klimstra et al., 2014; Lau & Marsee, 2013), bullying (Baughman et al., 2012), moral disengagement (Sijtsema et al., 2019), violence, and criminal behavior (Essau et al., 2006; Frick & Morris, 2004). People are surprisingly accurate in their assessments of others’ antisocial traits, and are significantly more accurate about others than themselves via self-report measures (Oltmanns & Turkheimer, 2009).

In two blocks of trials, participants made two sets of decisions, choosing which of two differently motivated (aesthetic vs. functional) but otherwise-matched characters was more likely to behave in a compassionate way towards others, and which was more likely to behave in a selfish and manipulative way towards others. We predicted that the aesthetically-motivated character would be chosen as more compassionate and less selfish/manipulative towards others, compared to the matched functionally-motivated character. We predicted that this would be true across all activities, including formal artforms (music, painting) as well as other everyday activities that may be aesthetically motivated by beauty (cooking, being in nature, exercising, doing math). Alternatively, if people believe that any engagement in conventional art activities provides evidence of prosociality, then they should make different judgments about peoples’ prosociality and antisociality on the basis of activity type (conventional art forms vs. activities not considered conventional art forms), and should make similar social judgments for anyone engaging in an activity regardless of aesthetic vs. functional motivations. Methods and planned analyses were preregistered at https://osf.io/gmey5/?view_only=afd333b8c1574d428134c9ed5f21658c and all materials can be found at https://osf.io/gmey5/?view_only=281399e422cb4181a2fbacfdd077ebcb.

### Method

#### Participants.

As preregistered, *N* = 360 undergraduates (*n* = 60 per activity condition) at a large public university in Southern California participated in the study online, in exchange for course credit (Mean age = 20.6 yrs, *SD* = 2.1 yrs., 72.8% female). This preregistered sample size was based on power analysis (80% power of detecting a 0.075 proportion difference vs. chance). Additional participants were tested and their data excluded based on preregistered exclusion criteria: failing when asked to select the number “two” (*n* = 6); choosing a pebble as more compassionate than an adult human (*n* = 45); or choosing a pebble as more selfish/manipulative than an adult human (*n* = 9).

#### Procedure and Design.

**Figure 2.**
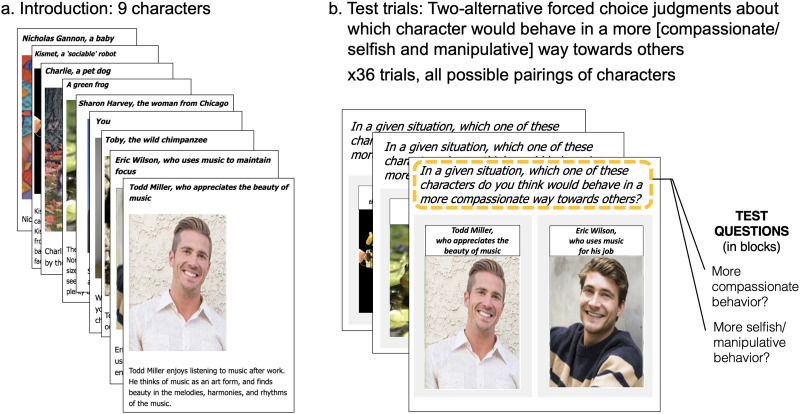
Methods, Experiment 1. Participants were introduced to nine characters, including distractors to avoid demand effects, along with two critical matched characters: A pair of adults engaging in the same activity, one motivated by the aesthetic value of the activity, and the other motivated by the functional usefulness of the activity. On each of 36 trials, participants saw two characters from this set, and selected which one of the pair would behave in a more compassionate, or selfish and manipulative, way towards others.

Participants were introduced to nine characters in random order, each depicted via a photographic image and a short vignette. Seven of these characters were distractors, aimed to disguise the purpose of the study, and embedded amongst them we included two characters that served as our manipulation of interest. Both characters in this critical pair were described as doing the same activity but for different reasons (see Stimuli). In the test phase of the experiment, participants were presented with each possible pairing of the nine characters separately (with images and abbreviated descriptive phrases) and were asked to make forced-choice pairwise comparisons between characters. There were 36 pairs in total, which were presented in random order, with left-right positions randomized (see Figure 2).

Participants made the full set of comparisons twice, answering a different test question in each block: “In a given situation, which one of these characters do you think would behave in a more [compassionate] [selfish and manipulative] way towards others?” The order of the blocks was randomized. Participants then completed two attention checks (see Participants), answered two free response questions asking what they thought the study was about, and chose which of our two critical characters they thought was most similar to them^1^. Finally, they filled out a standard demographics survey with questions about their age, gender, race, and ethnicity. The median duration of the experiment was 13.4 minutes.

#### Stimuli.

Our seven distractor characters were taken from Gray et al. (2007, images provided by K. Gray) and included a frog, pet dog, monkey, human baby, a neutral human adult (always of the opposite gender to the critical characters), a sociable robot, and “You” (participants were shown a picture of a mirror and asked to consider themselves). The remaining two characters were the manipulation of interest: Both were adults of the same gender, described as engaging in the same activity (e.g., listening to music); One character was described as being motivated by the aesthetic beauty of the activity, while the other was described as being motivated by the usefulness of that activity in achieving some functional goal. We created man and woman versions of all our critical character-pairs, and counterbalanced across participants whether participants saw the pair of men vs. women.

Across six between-subject conditions (*n* = 60 for each), we varied the activities that the critical pair of characters engaged in: listening to music, painting, cooking food, being in nature, exercising, or doing math (see Table 1). The image and name that was assigned to the aesthetically- vs. functionally-motivated character was counterbalanced across participants, such that half of participants saw the aesthetically-motivated character depicted as one image/name, and the other half of participants saw the aesthetically-motivated character depicted as the other image/name. Within each critical character-pair, descriptions were tightly matched for length, style, and type of information provided. Both characters were described as experiencing enjoyment and finding value in the activity; the aesthetically-motivated character enjoyed the activity itself, while the functionally-motivated character enjoyed some external outcome of the activity, which varied across activities.

**Table 1.** Descriptions of Critical Characters, Experiment 1

**Activity**	**Aesthetically-motivated Character**	**Functionally-motivated Character**
*Listening to music (28 words each)*	[Erin Wilson / Tina Miller // Eric Wilson / Todd Miller] enjoys listening to music after work. S/he thinks of music as an art form, and finds beauty in the melodies, harmonies, and rhythms of the music.	[Tina Miller / Erin Wilson // Todd Miller / Eric Wilson] uses music as part of his/her job. S/he uses it as a tool to maintain his focus, and enjoys how it helps her/him get work done.
*Painting (26 words each)*	[Erin Wilson / Tina Miller // Eric Wilson / Todd Miller] enjoys painting after work. S/he thinks of painting as an art form, and finds beauty in the colors, composition, and designs of the paintings.	[Tina Miller / Erin Wilson // Todd Miller / Eric Wilson] is a professional painter. S/he thinks of painting as a useful way to make a living, and enjoys it as a means of income.
*Cooking food (29 words each)*	[Erin Wilson / Tina Miller // Eric Wilson / Todd Miller] cooks a lot because s/he enjoys it and thinks of it as an art form. S/he finds beauty in the tastes, flavors, and textures of the food.	[Tina Miller / Erin Wilson // Todd Miller / Eric Wilson] cooks a lot as part of her/his everyday life. S/he uses it as a way to obtain energy and enjoys getting nutritious meals when s/he is hungry.
*Being in nature (29 words each)*	[Erin Wilson / Tina Miller // Eric Wilson / Todd Miller] enjoys spending time in nature during her/his free time, after work. S/he appreciates the beauty of the mountain vistas, the natural scenery, and the colors of sunsets.	[Tina Miller / Erin Wilson // Todd Miller / Eric Wilson] spends time in nature as part of her/his job. S/he enjoys her/his job. S/he often needs to walk through the woods to get to various work locations.
*Exercising (26 words each)*	[Erin Wilson / Tina Miller // Eric Wilson / Todd Miller] exercises every day because s/he enjoys it. S/he thinks of long-distance running as beautiful and sublime, an art form just like music or poetry.	[Tina Miller / Erin Wilson // Todd Miller / Eric Wilson] exercises a lot because of her/his job as a professional trainer. S/he uses it as a tool and enjoys how it maintains her/his fitness.
*Doing math (27 words each)*	[Erin Wilson / Tina Miller // Eric Wilson / Todd Miller] enjoys doing math in her/his free time, after work. S/he thinks of math as beautiful and elegant, an art form just like music or poetry.	[Tina Miller / Erin Wilson // Todd Miller / Eric Wilson] does math as part of her/his job. S/he uses it as a tool for solving problems and getting things done at work, which s/he enjoys.

*Note*. Character pairs always included adults described as enjoying the same activity; One character was motivated by the aesthetic beauty of the activity, while the other was motivated by the usefulness of that activity in achieving some functional goal, which varied across activities. Descriptions were tightly matched for length and style, and character images/names/genders were counterbalanced across participants.

### Results

**Figure 3.**
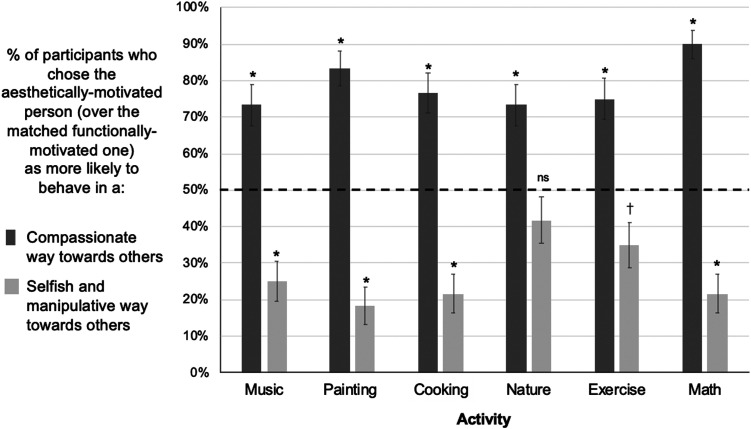
Results, Experiment 1. On critical test trials that directly pitted aesthetically-motivated characters against matched functionally-motivated characters doing the same activity, participants chose the aesthetically-motivated characters as behaving more compassionately and less selfishly/manipulatively towards others. * *p*-values below Bonferroni-corrected alpha level of 0.05/6 or 0.0083, † *p*-values below 0.05, ns: *p*-values above 0.05, two-tailed binomial tests versus 50% chance.

On the critical trial where participants were asked to choose which of the matched characters they thought would behave in a more compassionate way towards others, 78.61% of participants selected the aesthetically-motivated character as more likely to behave compassionately towards others than the matched functionally-motivated character (283/360, *p* < .001, Odds Ratio [OR] = 3.68, binomial test vs. chance proportion of 50%). The type of activity was not a significant predictor of participants’ tendency to choose the aesthetically-motivated character (*F* = 1.77, *p* = .115, nested logistic regression; Full model: predicts participants’ choice of aesthetically- vs. functionally-motivated character from predictors of activity type and gender of the character pair; Simpler model: same as the full model, but excludes activity type as a predictor). The gender of the character pair was also not a significant predictor of participants’ judgments (*F* = 2.05, *p* = .152, nested logistic regression; Simpler model: same as the full model, but excludes characters’ gender as a predictor). Participants reliably chose the aesthetically- motivated character as more likely to behave compassionately than the functionally-motivated character across all six of the activities tested, even with a conservative correction for multiple comparisons (Bonferroni-corrected alpha = .0083): Listening to music (44/60, 73.3%, *p* < .001, OR = 2.75), painting (50/60, 83.3%, *p* < .001, OR = 4.99), Cooking (46/60, 76.7%, *p* < .001, OR = 3.29), being in nature (44/60, 73.3%, *p* < .001, OR = 2.75), exercising (45/60, 75.0%, *p* < .001, OR = 3.00), and doing math (54/60, 90.0%, *p* < .001, OR = 9.00; see Figure 3).

In contrast, when asked to select who was more likely to behave in a selfish and manipulative way towards others, participants’ judgments flipped, such that they chose the aesthetically-motivated character *less* often than the functionally-motivated one (27.2% choosing the aesthetically-motivated character, 98/360, *p* < .001, OR = .37, binomial test vs. chance proportion of 50%). For these anti-social judgements, the kind of activity performed was a significant predictor (*F* = 2.46, *p* = .031, nested logistic regression, see above), while gender of the character pair again was not a significant predictor (*F* = 1.45, *p* = .228). Participants selected the aesthetically-motivated character significantly less often for all but two of the activities (when using a Bonferroni-corrected alpha of .0083), with “being in nature” and “exercising” as the exceptions (Listening to music: 15/60, 25.0%, *p* < .001, OR = .33; Painting: 11/60, 18.3%, *p* < .001, OR = .22; Cooking: 13/60, 21.7%, *p* < .001, OR = .28; Exercising: 21/60, 35.0%, *p* = .027, OR = .54; Doing math: 13/60, 21.7%, *p* < .001, OR = .28; Being in nature: 25/60, 41.7%, *p* = .245, OR = .72; see Figure 3).

#### Demand Effects Check: Most Participants Remained Unaware of Hypothesis.

Free-response answers regarding what the study was about were coded by two independent raters (unaware of condition and other responses) for mentions of the critical characters, including mentions of the specific activity of interest (e.g., music, painting, cooking), or mentions of a manipulation of the characters’ hobbies or occupations (as this feature did not appear in distractor characters). Inter-rater reliability was 100%. Only 6/360 participants (1.67%) mentioned the critical characters when asked what the study was about (1/360 mentioning the activity of interest, 5/360 mentioning hobbies or occupations). As pre-registered, results reported include these data points. All findings remain the same whether these participants’ data are included or excluded, with no differences in conclusions or statistical significance.

#### Manipulation Check: Participants Made Sensible Judgments About Distractor Characters.

**Figure 4.**
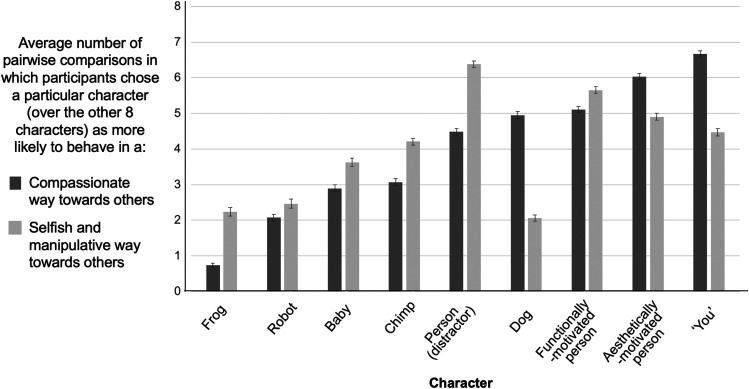
Character Rankings, Experiment 1. Character rankings, Experiment 1. Participants made sensible choices about which of the characters was more likely to behave in a compassionate way or selfish and manipulative way towards others. The Y-axis shows the average number of pairwise comparisons in which participants chose a particular character over the other eight characters. Error bars are standard errors.

To check our manipulation and probe the validity of the task as a measure of social judgments, we examined participants’ judgments across the full range of characters, including the distractors as well as the critical characters. As pre-registered, we calculated the total number of times that a particular character was selected as more likely to behave compassionately or more selfishly/manipulatively (Figure 4) out of the eight pairwise comparisons between that character and every other character. We looked at this data in aggregate, collapsed across all conditions. Overall, participants made sensible judgments, rating the frog, robot, and human baby as least likely to behave compassionately towards others, and the dog, frog, and robot as least likely to behave selfishly/manipulatively towards others. The adult human characters were ranked highly likely to behave compassionately toward others, as was the dog (who was described as a pet), with the self ranked most highly. The adult human characters were also ranked most likely to behave selfishly towards others, with the self notably ranked below the other adult human individuals.

### Discussion

In the first experiment, we found that participants viewed characters who were aesthetically-motivated as significantly more likely to behave compassionately toward others than those who engaged in the same activity achieve a non-aesthetic, functional goal. We also found that participants’ choices flipped when asked about anti-social characteristics, such that aesthetically-motivated characters were chosen as selfish and manipulative significantly *less* often than functionally-motivated characters. These effects held true across multiple different pairs of stimuli and activities, and irrespective of characters’ images, names, and genders, suggesting that our results are not driven by idiosyncratic aspects of any particular stimulus. These results also are not explained by demand characteristics: Participants were successfully distracted by the irrelevant characters, and did not realize that the aesthetic vs. functional motivations were of primary interest.

Notably, we found effects with roughly equivalent effect sizes across a wide variety of everyday activities, including not only formal artforms like listening to music and painting, but also activities that are not traditionally thought of as involving aesthetic engagement, such as cooking food, being in nature, exercising, and doing math. This provides evidence that participants are not making positive social attributions based on simply knowing that someone engaged in formal arts activities, for example. Instead, participants’ prosocial attributions appear to be driven by evidence of others’ aesthetic motivations, the tendency to value an activity for its own sake rather than as a means to an end.

Only in two cases were participants indifferent between aesthetically- and functionally-motivated characters: when asked to choose which of two characters who enjoyed [being in nature/exercising] was more likely to behave in a selfish and manipulative way towards others. If this effect replicates (see Exp. 3), this finding raises the question of whether certain activities, like being in nature or exercising, are seen as having the power to decrease antisocial behavior regardless of why they are undertaken. For example, people may have the intuitive theory that being in nature increases positive emotions and prosocial behavior for anyone who engages in it, as this has been found true in experimental work (Dopko et al., 2019; Mayer et al., 2009).

## EXPERIMENT 2

We next probe *why* aesthetic motivations impact attributions of others’ prosocial and antisocial qualities, testing our hypothesis that this effect is mediated by inferences about others’ broader mental capacities, particularly their emotional sensitivity. When perceiving and representing others’ minds, our attributions can be mapped on to a small number of major components. These are often termed the capacity for *experience*, the ability to experience physical and emotional sensations like pain or joy; and *agency* or intelligence, the ability to think complex thoughts, make decisions, and produce controlled behaviors (Gray et al., 2007; Knobe & Prinz, 2008; Robbins & Jack, 2006; Sytsma & Machery, 2012; see Weisman et al., 2017 for an alternative 3-factor framework). Inferences about these mental capacities have important social consequences: They directly impact decisions such as how morally wrong it would be to harm someone, or how responsible someone is for their actions (Goodwin, 2015; Gray et al., 2007).

When a person learns that someone is engaging in an activity for its intrinsic value, for example because they are motivated by its beauty, this may drive broader inferences about their enhanced capacity for emotionality (Agrawal et al., 2023; Larsen & Sackris, 2020; Menninghaus et al., 2019). This belief in higher emotional sensitivity may drive judgments about their likelihood to behave prosocially (Gray et al., 2007; Sytsma & Machery, 2012). Alternatively, it is also possible that the distinct behavioral predictions we found in Exp. 1 are a limited phenomenon, without broader consequences for reasoning about others. In this case, we would expect that aesthetic vs. functional motivations do not lead to differences in attributions of others’ mental capacities, such as their capacity for experience (whether emotional, or physical) or their intelligence. Lastly, another possibility is that people may view aesthetically-motivated others as having a richer mental life in general, with more emotions, sensations, and cognitive abilities of every kind. If so, then instead of making a selective inference about emotional sensitivity, people should additionally attribute more physical sensitivity (the capacity for physical sensations, like hunger or pain), as well as greater intelligence to those motivated by intrinsic aesthetic value.

Here, we tease apart these accounts, building on the method used in Exp. 1, which was designed to minimize demand effects and provide tight control for the factors of interest. Participants again made pairwise, forced-choice judgments about a set of characters, including a pair of matched characters who engage in the same activity, one for aesthetic and one for functional reasons. In one block of trials, participants judged which character in each pair was more emotionally sensitive. In two other randomly-ordered blocks of trials, participants were asked which character in each pair was more intelligent, or more able to feel physical sensations, like pain, pleasure, and hunger.

We predicted that participants would attribute greater emotional sensitivity to the aesthetically-motivated characters than the matched functionally-motivated characters. We further hypothesized that this attribution would not extend to judgements of intelligence, a distinct component of mind attribution. Our predictions for physical experience capacities were less clear, as the capacity for emotional experience and physical experience are highly correlated (Agrawal et al., 2023; Gray & Wegner, 2012; Piazza et al., 2014). If aesthetically-motivated others are seen as having a higher capacity for emotional experience, as hypothesized, then this may lead to the broader inference that aesthetically-motivated others have a higher capacity for physical experience as well.

As in Exp. 1, our methods and analyses were pre-registered (https://osf.io/gmey5/?view_only=afd333b8c1574d428134c9ed5f21658c), and can be found at https://osf.io/gmey5/?view_only=281399e422cb4181a2fbacfdd077ebcb.

### Method

#### Participants.

Undergraduates at a large public university in Southern California (*N* = 360, *n* = 60 per activity condition) participated in an online experiment for course credit (Mean age = 20.5 yrs, SD = 2.3 yrs., 78.9% female). The sample size was decided *a priori* based on the power analysis from Exp. 1. Data from 21 additional participants was collected but excluded based on preregistered exclusion criteria: choosing a pebble over an adult human as being more emotional, intelligent, or able to feel physical sensations like pain/pleasure/hunger (*n* = 13); failing when asked to select the number “two” (*n* = 5); or facing technical issues on the online survey platform, identified by participants’ answers to a question about whether technical difficulties occurred (*n* = 4; e.g., images or answer choices not loading, or accidentally being shown multiple counterbalance conditions).

#### Procedure and Stimuli.

The procedure and stimuli were identical to those of Exp. 1, except that participants now completed three sets of pairwise judgments for all character pairs (36 trials each). Each of the three randomly-ordered blocks measured a different mental capacity: “Which one of these characters do you think is more [emotionally sensitive / intelligent / able to feel physical sensations like pain, pleasure, and hunger]?”

In addition, the description of the functionally-motivated character in the Cooking condition was modified to avoid explicit mention of hunger, as one of the test questions involved judging the characters’ capacity for physical sensations like hunger. The new vignette read: “[e.g., Tina Miller] cooks a lot as part of her everyday life. She uses it as a way to obtain energy and enjoys getting healthy nutrition from her meals.”

This experiment took participants 18.4 minutes to complete, on average.

### Results

**Figure 5.**
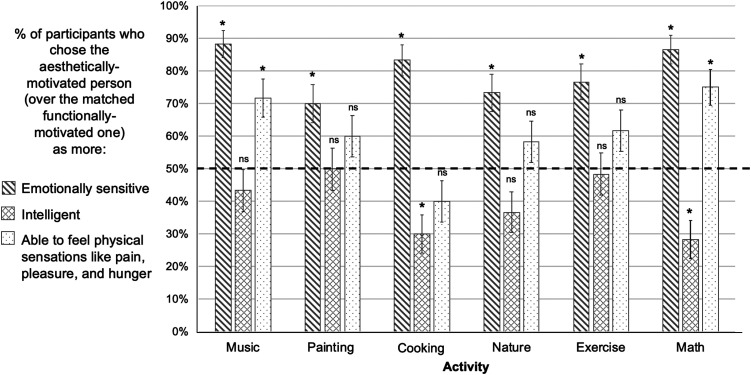
Results, Experiment 2. *Note*. On the critical test trials that directly pitted aesthetically-motivated characters against matched functionally-motivated characters doing the same activity, participants reliably chose the aesthetically-motivated characters as more emotionally sensitive. In contrast, they did not judge aesthetically-motivated characters to be more intelligent, and judged aesthetically-motivated characters to have a greater capacity for physical experience for some activities and not others. * *p*-values below Bonferroni-corrected alpha level of 0.05/6 or 0.0083, † *p*-values below 0.05, ns: *p*-values above 0.05, two-tailed binomial tests versus 50% chance.

Across all activities, we saw robust effects of our manipulation on perceived emotionality of the characters: On the critical trial pitting the two matched characters, 79.7% of participants selected the aesthetically-motivated character as being more emotionally-sensitive than the functionally-motivated character (287/360, *p* < .001, OR = 2, binomial test vs. chance proportion of 50%). Nested logistic regressions revealed that there were no significant effects of either the character’s gender (*F* = .018, *p* = .894) or the activity they were doing (*F* = 2.11, *p* = .061). In line with this, participants reliably selected the aesthetically-motivated character as more emotionally sensitive across each of the six activities (Bonferroni-corrected alpha = .0083; Listening to music: 53/60, 88.3%, *p* < .001, OR = 7.57; Painting: 42/60, 70%, *p* = .0027, OR = 2.33; Cooking: 50/60, 83.3%, *p* < .001, OR = 5.00; Being in nature: 44/60, 73.3%, *p* < .001, OR = 2.75; Exercising: 46/60, 76.7%, *p* < .001, OR = 3.29; Doing math: 52/60, 86.7%, *p* < .001, OR = 6.50; see Figure 5).

As predicted, the emotionality findings were specific: We did not find the same pattern of selections for intelligence judgments. Only 39.4% of participants (significantly *fewer* than chance) judged the aesthetically-motivated character as being more intelligent than the functionally-motivated one (142/360, *p* < .001, OR = .65, binomial test vs. chance proportion of 50%). Nested logistic regressions revealed no significant overall effect of activity (*F* = 2.20, *p* = .052). However character gender was a significant predictor of intelligence judgments (*F* = 5.82, *p* = .016): Holding all other variables constant, the odds of a participant choosing the aesthetically-motivated character as more intelligent than the functionally-motivated character was increased by 70% (95% CI [.10, 1.62]) for men versus women characters.

Looking individually at each of the six activities, aesthetically-motivated characters were never reliably selected as higher in intelligence than functionally-motivated characters (Bonferroni-corrected alpha level = .0083; Listening to music: 26/60 chose aesthetically-motivated characters as being more intelligent, 43.3%, *p* = .366, OR = .76; Painting: 30/60, 50%, *p* = 1, OR = 1.00; Cooking: 18/60, 30%, *p* = .0027, OR = .43; Being in nature: 22/60, 36.7%, *p* = .052, OR = .58; Exercising: 29/60, 48.3%, *p* = .90, OR = .94; Doing math: 17/60, 28.3%, *p* = .0011, OR = .40; note that low *p*-values here indicate that people selected aesthetically-motivated characters significantly *less* often than chance; see Figure 5).

People also judged the aesthetically-motivated character as more capable of experiencing physical sensations than the functionally-motivated character (220/360, 61.1%, *p* < .001, OR = 1.57, binomial test vs. chance proportion of 50%). Nested logistic regression analyses revealed no effect of character gender (*F* = .445, *p* = .505), and a significant effect of activity (*F* = 3.85, *p* = .0017). The overall effect on capacity for physical experience was driven primarily by two activities, listening to music (43/60, 71.7%, *p* = .0011, OR = 2.53), and doing math (45/60, 75%, *p* < .001, OR = 3.00). Across other activities, people’s judgements did not differ from chance (Painting: 36/60, 60%, *p* = .155, OR = 1.5; Cooking: 24/60, 40%, *p* = .155, OR = .67; Being in nature: 35/60, 58.3%, *p* = .245, OR = 1.40; Exercising: 37/60, 61.7%, *p* = .092, OR = 1.61; Bonferroni-corrected alpha level = .0083; see Figure 5).

#### Demand Effects Check: Most Participants Remained Unaware of Hypothesis.

Free response answers asking participants what the study was about were coded in the same way as Exp. 1 (inter-rater reliability = 100%). Six of the 360 participants (1.67%) mentioned the critical characters when asked what the study was about (4/360 mentioning the activity of interest / critical characters specifically, 2/360 mentioning characters’ hobbies or occupations). All findings remain the same whether these participants’ data are included or excluded, with no differences in conclusions or statistical significance. As preregistered, results reported include these data points.

#### Manipulation Check: Judgments of Distractor Characters Match Established Findings.

**Figure 6.**
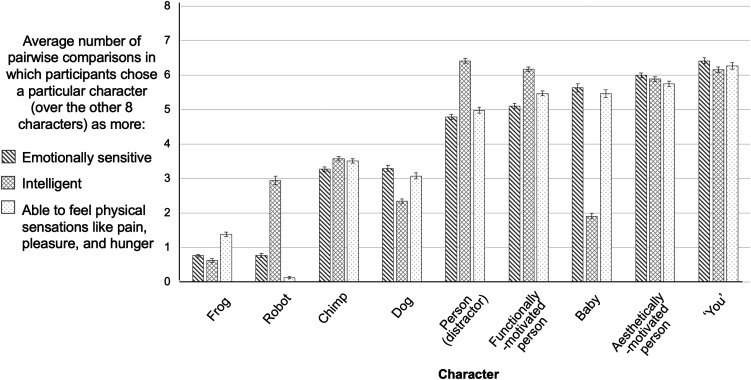
Character Rankings of Mental Traits, Experiment 2. *Note*. Participants made sensible choices about which of the characters was more emotionally sensitive, more intelligent, and more able to feel physical sensations like pain, pleasure, and hunger. The Y-axis shows the average number of pairwise comparisons in which participants chose a particular character over the other eight characters. Error bars are standard errors.

To check our manipulation and probe the validity of the task as a measure of social judgments, we examined participants’ judgments regarding the distractor characters (as well as critical characters) as pre-registered, in the same way as in Exp. 1. Rankings broadly replicated the patterns in previous work where people made similar two-alternative forced choices between similar characters’ mental capacities (Gray et al., 2007, see Figure 6). For example, babies were ranked as high in emotional and physical experience capacities but low in intelligence, while the robot was ranked as higher in intelligence but low in the capacities for emotional or physical experience, and adult human characters were always ranked highest on all mental capacities.

### Discussion

In this experiment, our results provide empirical support for the first step of our hypothesized causal model (see Figure 1): Learning about others’ aesthetic vs functional motivations impacts people’s judgments about their broader mental capacities, particularly their capacity for emotionality. Across all activities and all stimulus conditions, people reliably chose characters who were aesthetically motivated—engaging in an activity because of its intrinsic value, or beauty—as being significantly more emotionally sensitive than matched characters doing the same activity to achieve some external, functional goal. This suggests that the effects seen in Exp. 1 are not limited to inferences about prosocial and antisocial behavior, but instead extend to broad trait-level judgments about others’ minds and mental capacities.

As predicted, we found that people were specific in their attributions: Participants did not judge aesthetically-motivated characters to be more intelligent than functionally-motivated ones. Thus, participants did not simply always select aesthetically-motivated characters; nor did they broadly judge aesthetically-motivated characters as having a richer mental life across all mental capacities. Instead, participants systematically judged that aesthetically-motivated characters were *less* intelligent than functionally-motivated characters, perhaps because functionally-motivated characters show goal-directed behavior, an important component of the behavior of intelligent, agentic entities (e.g., Baker et al., 2009, 2017). These systematic inferences about intelligence underline the surprisingly broad set of interpersonal judgements that are impacted by information about others’ aesthetic vs. functional motivations.

Finally, we found that participants broadly judged aesthetically-motivated characters to be more capable of physical sensations like pain, pleasure, and hunger than matched functionally-motivated characters. This result is somewhat surprising, as the capacity for physical experience seems notably distinct from the capacity for aesthetic appreciation. However, this connection is in line with previous work showing that judgments about others’ physical and emotional capacities are highly correlated (Agrawal et al., 2023; Eisenberger & Lieberman, 2004; Piazza et al., 2014). Thus, impacts on judgments of emotionality may lead to further impact on judgements of physical sensation. These findings were also primarily driven by two activities, rather than extending across all of them (music, math). We test whether this effect replicates as part of the next two experiments.

## EXPERIMENT 3

The first two experiments provide evidence for the two major sub-components of our model: Participants judged that people who were motivated to do an activity for aesthetic reasons would behave more prosocially, and less antisocially, than those with non-aesthetic, functional motivations (Exp. 1). In addition, participants inferred that aesthetically-motivated people were more emotionally sensitive (Exp. 2). Next, we test our hypothesis that these judgements are related. In particular, we hypothesize that inferences about others’ heightened emotionality mediate the link between observed aesthetic engagement and inferred prosocial dispositions.

To test this causal model, we gathered within-subject measures of judgments of prosocial behavior (“more likely to behave compassionately toward others”, as in Exp. 1), and broader mental capacities (“more emotionally sensitive / intelligent / able to feel physical sensations like pain, pleasure, and hunger”, as in Exp. 2), in order to characterize the relationship between these judgments. We tested two predictions of our causal model. First, we expected that participants’ choices about the characters’ emotionality would predict their judgements about compassionate behavior. Second, we expected that emotionality would be the best predictor of compassionate behavior judgments, better than judgements about intelligence and capacity for physical experience. It remains possible that intelligence and capacity for physical experience will also explain some variability in participants’ prosociality judgments: Previous work on mind perception has shown that intelligence and physical experience capacities strongly impact perceptions of others’ morality (Epley & Waytz, 2010; Gray et al., 2007; Knobe & Prinz, 2008; Robbins & Jack, 2006; Sytsma & Machery, 2012; Weisman et al., 2017), and our manipulation of aesthetic vs. functional motivation did impact inferences about these mental capacities in Exp. 2. However, we predicted that emotional sensitivity would be a stronger predictor than these two other capacities.

This study also provided an opportunity to test the replicability of findings, and their generalizability to a broader population. We shifted away from testing undergraduate students, and instead tested a more diverse population of adult participants with respect to age, geographical location, and ethnicity (recruited via Prolific, an online study pool extensively validated in previously published studies; Palan & Schitter, 2018; Peer et al., 2017). We expected to replicate our findings in this broader sample, such that aesthetically-motivated characters would again be chosen as more likely to behave compassionately, and be more emotionally sensitive, than matched functionally-motivated characters.

Preregistered stimuli, methods, and analyses were preregistered (https://osf.io/gmey5/?view_only=afd333b8c1574d428134c9ed5f21658c) and are available at https://osf.io/gmey5/?view_only=281399e422cb4181a2fbacfdd077ebcb.

### Method

#### Participants.

Adults residing in the United States (*N* = 360, Mean age = 35.89 yrs, *SD* = 12.58 yrs., 52.78% female) participated via the well-established Prolific online platform (e.g., Palan & Schitter, 2018). Participants came from all four regions of the country: Northeast, 21.2%; West, 19.5%; South, 40.7%; Midwest, 18.7% (of the 359/360 participants for whom latitude/longitude information was available; regions based on U.S. census groupings, U.S. Census Bureau, 2021). All participants were over age 18, had 93% or higher approval rate of prior work on the Prolific platform, and received $4.00 for participation (equivalent to $12/hour).

Data from 30 additional participants was collected and excluded because those participants failed one of our preregistered exclusion criterion (choosing a pebble over an adult human as being more emotional, intelligent, or able to feel physical sensations like pain/pleasure/hunger, *n* = 20; having technical difficulties, *n* = 10).

#### Procedure and Stimuli.

The procedure and stimuli were identical to Exp. 2, except that participants now completed four blocks of pairwise judgments across all character pairs (36 trials each). The first block always involved judgments about the characters’ prosociality (“In a given situation, which one of these characters do you think would behave in a more compassionate way towards others?”) Each of the three subsequent blocks was randomly-ordered, and each measured a different mental capacity (“Which one of these characters do you think is more [emotionally sensitive] [intelligent] [able to feel physical sensations like pain, pleasure, and hunger]?”). On average, the experiment lasted for 21.7 minutes.

### Results

#### Key Findings from Exps. 1 & 2 Replicate in a Broader Participant Sample.

**Figure 7.**
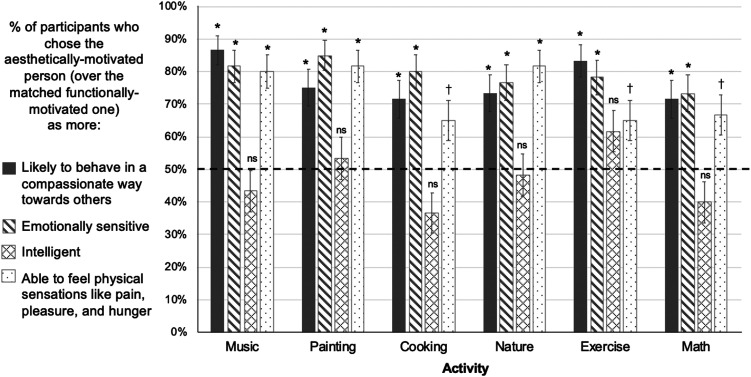
Results, Experiment 3, Using a Within-Subjects Design and Testing a Broader Participant Sample. *Note*. On critical test trials that directly pitted aesthetically-motivated characters against matched functionally-motivated characters doing the same activity, participants reliably chose the aesthetically-motivated ones as behaving more compassionately, more emotionally sensitive, and more able to feel physical sensations like pain, pleasure, and hunger. In contrast, participant judgments about the characters’ intelligence did not significantly vary based on whether the characters were aesthetically or functionally motivated. * *p*-values below Bonferroni-corrected alpha level of 0.05/6 or 0.0083, † *p*-values below 0.05, ns: *p*-values above 0.05, two-tailed binomial tests versus 50% chance.

On the critical trial that pitted the two matched characters, participants reliably selected the aesthetically-motivated character as more likely to behave compassionately than the matched functionally-motivated character (277/360, 76.9%, *p* < .001, OR = 3.34, binomial test vs. chance proportion of 50%). This effect held across each of the individual activities (all *p*’s = < 0.001; see Figure 7). Nested logistic regressions revealed no significant effects of activity (*F* = 1.51, *p* = .18), but there were significant effects of character gender (*F* = 4.64, *p* = .031) such that the odds of a participant choosing the aesthetically-motivated character as more likely to behave compassionately increased by 73% (95% CI [.05, 1.87]) for characters who were men versus women, holding all other variables constant.

Participants also selected aesthetically-motivated characters as more emotionally sensitive (285/360, 79.2%, *p* < .001, OR = 3.80, binomial test vs. chance proportion of 50%), across each of the six individual activities (all *p*’s < 0.001). Nested logistic regressions revealed no significant effects of activity (*F* = .61, *p* = .69), but character gender did have an impact (*F* = 4.94, *p* = .026) such that the odds of a participant choosing the aesthetically-motivated character as more emotionally sensitive increased by 78% (95% CI [.07, 2.03]) for characters who were men versus women, holding all other variables constant.

As in Exp. 2, participants did not reliably choose aesthetically-motivated characters as more intelligent than the functionally motivated characters; in contrast to Exp. 2, participants’ rate of choosing aesthetically-motivated characters was no lower than chance (170/360, 47.2%, *p* = .317, OR = .89, binomial test vs. chance proportion of 50%). This pattern of chance responding for intelligence judgements held true across all six individual activities (Listening to music: 26/60, 43.3%, *p* = .366, OR = .76; Painting: 32/60, 53.3%, *p* = .699, OR = 1.14; Cooking: 22/60, 36.7%, *p* = .0519, OR = .58; Being in nature: 29/60, 48.3%, *p* = .897, OR = .93; Exercising: 37/60, 61.7%, *p* = .093, OR = 1.61; Doing math: 24/60, 40%, *p* = .155, OR = .67; Bonferroni-corrected alpha level = .0083; see Figure 7). Nested logistic regressions revealed no significant effects of activity (*F* = 2.13, *p* = .058), but significant effects of character gender on intelligence judgments (*F* = 11.8, *p* < .001) such that the odds of a participant choosing the aesthetically-motivated character as more intelligent increased by 110% (95% CI [.37, 2.23]) for characters who were men versus women, holding all other variables constant.

Participants also selected the aesthetically-motivated character as having a higher capacity for physical experience than the functionally-motivated matched character, showing slightly larger effects than in the previous experiment (264/360, 73.3%, *p* < .001, OR = 2.75, binomial test vs. chance proportion of 50%). In this experiment, we also saw more consistent effects of our manipulation on physical experience across activities: P-values across all activities were below an alpha of 0.05, with music, painting, and being in nature reaching the conservative Bonferroni-corrected alpha of .0083 (Listening to music: 48/60, 80%, *p* < .001, OR = 4.00; Painting: 49/60, 81.7%, *p* < .001, OR = 4.46; Cooking: 39/60, 65%, *p* = .027, OR = 1.86; Being in nature: 49/60, 81.7 %, *p* < .001, OR = 4.46; Exercising: 39/60, 65%, *p* = .027, OR = 1.86; Doing math: 40/60, 66.7%, *p* = .014, OR = 2.00). Nested logistic regressions revealed a small but significant effect of activity (*F* = 2.28, *p* = .044) and no effect of character gender (*F* = .53, *p* = .47).

#### Model Comparison: Emotional Sensitivity Best Predicts Prosocial Behavior Judgements.

Using mixed-effects logistic regression, we found that emotional sensitivity, intelligence, and capacity for physical experience each were significant predictors when individually added to the null model predicting compassion judgments as a function of activity and stimulus gender (Emotionality: *F* = 71.63, *p* < .001; Intelligence: *F* = 25.98, *p* < .001; Physical Experience Capacities: *F* = 35.98, *p* < .001; nested model comparisons vs. a null model; see Table 2).

**Table 2.** Model Comparison Results, Experiment 3

**Predictors of Compassionate Behavior**	**AIC Value of Model (lower implies better fit)**	**Nested Model Comparisons vs. first model**	
		***F***	***p***
Activity, Gender	390.67	–	–
Emotional Experience, Activity, Gender	321.05	71.63	<.001
Intelligence, Activity, Gender	366.69	25.98	<.001
Physical Experience, Activity, Gender	356.70	35.98	<.001
Emotional Experience, Intelligence, Activity, Gender	299.09	47.79	<.001
Emotional Experience, Physical Experience, Activity, Gender	308.54	43.07	<.001
Intelligence, Physical Experience, Activity, Gender	333.62	30.52	<.001
Emotional Experience, Intelligence, Physical Experience, Activity, Gender	285.13	37.18	<.001

To assess which combination of mental capacity predictors best explained compassion judgments, we conducted a series of model comparisons using AIC values, estimates of out-of-sample prediction error that are often used as a measure of model fit in comparative model selection (Wagenmakers & Farrell, 2004). Using AIC as a measure of model fit, with lower AIC values indicating better-fitting models, the model including emotionality (*AIC* = 321.05) was the best predictor of compassion judgments, compared to both the model including intelligence (*AIC* = 366.69; *p* < .001, analysis of Akaike weights to test whether emotionality model has a significantly lower AIC) and the model including physical experience (*AIC* = 356.70; *p* < .001, analysis of Akaike weights to test if emotionality model has a significantly lower AIC).

Next, we looked for the combination of mental capacities that best explained compassion judgments. All three mental capacities provided some amount of unique predictive power such that the full model, including emotionality, intelligence, and physical experience capacities as predictors, best fit the data (*AIC* = 285.13; *p* < .001, analysis of Akaike weights to test whether the full model has a significantly lower AIC than any other smaller model containing a subset of the mental capacities; see Table 2).

#### Demand Effects Check: Most Participants Remained Unaware of Hypothesis.

Free response answers asking participants what they thought the study was about were coded in the same way as Exp. 1 and 2. Three out of 360 participants mentioned the critical characters specifically, while two others noticed that we had manipulated characters’ hobbies or occupations (inter-rater reliability = 100%). All findings remain the same whether these participants’ data are included or excluded, with no differences in conclusions or statistical significance. As preregistered, results reported include these data points.

### Discussion

Overall, we found supportive evidence for our hypothesized causal model that aesthetic motivations impact judgments about others’ prosociality by signaling a heightened capacity for emotionality. We find that these inferences occur together: Emotionality judgments significantly predicted whether participants chose the aesthetically- or functionally-motivated characters as behaving more compassionately. As predicted, emotionality was the strongest predictor of compassionate behavior judgments. In addition, the other two mental capacities, intelligence and physical experience capacities, also each contributed unique predictive power.

We also successfully replicated our findings from Exp. 1 and 2 in a broader sample of participants from a wider range of ages and geographic locations across the U.S. Participants judged aesthetically-motivated characters as significantly more compassionate and emotionally sensitive, but not more intelligent than matched functionally-motivated characters. These findings were robust, and occurred across all activities–whether listening to music, painting, cooking, exercising, walking in nature, or doing math.

Participants also judged aesthetically-motivated characters as being more capable of experiencing physical sensations like pain, pleasure, and hunger, across all activities. This finding was more pronounced than in Exp. 2, where effect sizes (odds ratios) for all activities were smaller. In this experiment, all activities showed effects at an alpha of 0.05, and three activities passed the more conservative Bonferroni-corrected alpha cutoff (listening to music, painting, and being in nature) compared to only two in Exp. 2 (listening to music, doing math). This difference between findings may be explained by the differing populations across experiments: In Exp. 2, we tested undergraduate students at a large public university in Southern California. Contrastingly, the participant pool for Exp. 3 included a more diverse group, particularly with respect to age (not limited to undergrads) and geographic location (all over the U.S., see Participants for details).

We find a smaller effect size of our manipulation on the capacity for physical experience, compared to a larger effect of our manipulation on the capacity for emotional experience. This suggests that whether a person’s motivations are aesthetic vs. functional may be less informative about the capacity to experience physical sensations like pain and hunger than the capacity to experience emotional sensations. One result of the smaller effect sizes for physical experience is that our studies have lower power for this measure, and may be underpowered to reliably estimate the impact of our manipulation on physical experience at the level of each individual activity tested. This may also explain the differences between findings across experiments on this measure. Future work should replicate this finding with larger samples, to provide a clear estimate of the size of the effect of aesthetic motivation on physical experience capacities.

## EXPERIMENT 4

In the previous experiments, we found that aesthetic (vs. functional) motivations impacted a broad range of social attributions, leading to expectations of more compassionate behavior, less selfish and manipulative behavior, and greater emotional and even physical sensitivity. These attributions were at least somewhat selective: Aesthetically- motivated people were not seen as more intelligent than matched others, and were in some cases reliably seen as less intelligent.

What is the scope of the impact of aesthetic motivations on social attributions? Two plausible theories remain. First, it is possible that observing aesthetic motivations leads to a general expectation of increased prosocial behavior. In this case, observers should predict that someone with aesthetic motivations would always, on average, act more prosocially than a matched other person without aesthetic motivation, for any sort of prosocial behavior.

Prosocial behaviors can be defined as actions that intentionally promote others’ welfare or goals, particularly when these actions are effortful, time-consuming, or incur other costs (Hamlin et al., 2013; Pfattheicher et al., 2022; Powell, 2022). This diverse set of behaviors includes generosity, cooperation, altruism, and helpfulness, with helpfulness long playing a prominent role in work on prosociality (Callaghan et al., 2011; Dovidio, 1984; Hamlin et al., 2007; Penner et al., 2005). If aesthetic appreciation leads to a general expectation of increased prosocial behavior, then aesthetically-motivated people would be expected to be more helpful, in addition to behaving more compassionately.

Alternatively, aesthetic motivation may not lead to an expectation of increased helpfulness, in spite of an expectation for more compassionate behavior. The ability to be helpful towards others requires more than just the intent to be helpful: It also requires the agency, skill, and intelligence to help effectively (Dovidio, 1984). As such, which person is perceived as more helpful is impacted not just by their motivation to act prosocially, but also by their intelligence. In the previous experiments, we found that aesthetically-motivated people are not perceived as more intelligent, and in some cases are seen as less intelligent than functionally-motivated individuals. If observers’ behavioral predictions about aesthetically-motivated people are mediated by broader attributions of mental capacities (including the capacity for experience, and intelligence), then aesthetic appreciation may not reliably increase inferences about helpfulness, in spite of reliably increasing expectations of compassionate behavior.

To distinguish between these two accounts, here we test whether observers expect aesthetically-motivated individuals to behave in a more helpful way toward others than those without aesthetic motivations. In addition, we test whether and how predictions of helpful behavior are predicted by broader attributions of mental capacities, looking for the combination of these predictors that best explain compassion judgments. We hypothesized that attributions of intelligence would best explain judgements about helpful behavior, unlike judgements of compassionate behavior (for which emotional sensitivity was the best predictor).

To provide a tightly-controlled comparison of judgments about helpful vs. compassionate behavior, our measure of prosociality exactly mirrored the wording from previous experiments, with the crucial insertion of the word ‘helpful’ in place of ‘compassionate’. The measure thus became: “In a given situation, which one of these characters do you think would behave in a more helpful way towards others?”

Again, all stimuli, methods, and analyses were preregistered (https://osf.io/gmey5/?view_only=afd333b8c1574d428134c9ed5f21658c), and can be found at https://osf.io/gmey5/?view_only=281399e422cb4181a2fbacfdd077ebcb.

### Method

#### Participants.

Participants were undergraduates at a large public university in Southern California, who participated for course credit (*N* = 360, Mean age = 20.6 yrs, SD = 2.6 yrs., 75.3% female). Data from 44 additional participants was collected but excluded because those participants failed at least one of our preregistered exclusion criteria (choosing a pebble over an adult human as being more helpful, emotional, intelligent, or capable of feeling physical sensations like pain/pleasure/hunger, *n* = 38; failing when asked to select the number “two”, *n* = 6).

#### Procedure and Stimuli.

The procedure and stimuli were identical to Exp. 3, except that the first block of trials now involved judgments about the characters’ helpfulness (“In a given situation, which one of these characters do you think would behave in a more helpful way towards others?”). As in Exp. 2, each of the three subsequent blocks was randomly-ordered, and each measured a different mental capacity (“Which one of these characters do you think is more [emotionally sensitive] [intelligent] [able to feel physical sensations like pain, pleasure, and hunger]?”). The average duration of the experiment was 22.3 minutes.

### Results

**Figure 8.**
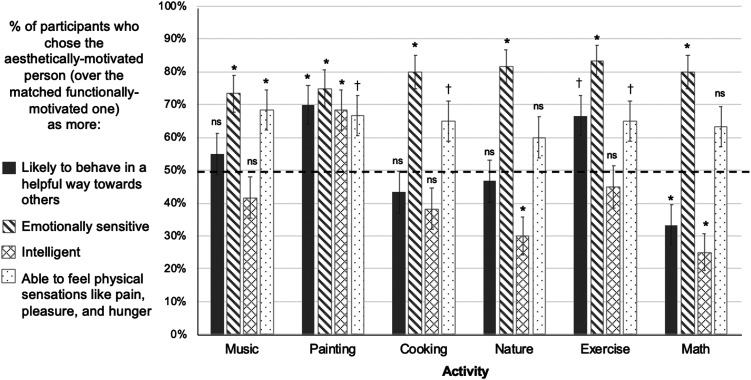
Results, Experiment 4. *Note*. On critical test trials that directly pitted aesthetically-motivated characters against matched functionally-motivated characters doing the same activity, participants reliably chose the aesthetically-motivated ones as more emotionally sensitive. Judgments about how helpful the characters would be towards others, as well as judgments about their intelligence and their capacity to feel physical sensations all varied based on activity. Overall, judgments about helpfulness were not significantly different from chance, and people judged aesthetically-motivated characters as being less intelligent but more capable of physical experience than matched functionally-motivated characters. * *p*-values below Bonferroni-corrected alpha level of 0.05/6 or 0.0083, † *p*-values below 0.05, ns: *p*-values above 0.05, two-tailed binomial tests versus 50% chance.

Overall, participants did not reliably select the aesthetically-motivated characters as behaving in a more helpful way than matched functionally-motivated characters (189/360, *p* = .37, OR = 1.11, binomial test vs. chance proportion of 50%). However, there were significant differences based on activity (*F* = 4.92, *p* < .001, nested model comparison). Aesthetically-motivated characters were reliably judged to be more helpful than the functionally-motivated characters when the activity was painting (42/60, 70%, *p* = .003, OR = 2.33) or exercising (40/60, 66.7%, *p* = .014, OR = 2.00). In contrast, they were reliably judged to be less helpful when the activity was doing math (20/60, 33.33%, *p* = .001, OR = .50). For other activities, participants’ choices did not differ from chance (Listening to music: 33/60, 55%, *p* = .519, OR = 1.22; Cooking: 26/60, 43.3%, *p* = .367, OR = .76; Being in nature: 28/60, 46.7%, *p* = .699, OR = .88; Bonferroni-corrected alpha level = .0083; see Figure 8). There were no differences based on character gender.

#### Key Findings Regarding Attributions of Broader Mental Capacities Replicate.

Participants again reliably selected aesthetically-motivated characters as being more emotionally sensitive than matched functionally-motivated characters (284/360, 78.9%, *p* < .001, OR = 3.74, binomial test vs. chance proportion of 50%). This effect held across all activities (all *p*’s < 0.001; see Figure 8). Overall, participants selected the aesthetically-motivated character as being significantly less intelligent, replicating effects from Exp. 2 (149/360, 41.4%, *p* = .0013, OR = .71, binomial test vs. chance proportion of 50%). Participants’ judgments about whether aesthetically- or functionally-motivated characters were more intelligent varied greatly based on activity (*F* = 4.92, *p* < .001, nested model comparison). Aesthetically-motivated characters were seen as less intelligent when the activity was being in nature (18/60, 30.0%, *p* = .003, OR = .43), and doing math (15/60, 25.0%, *p* < .001, OR = .33). In contrast, when the activity was painting, the aesthetically-motivated character was judged as more intelligent (41/60, 68.33%, *p* = .006, OR = 2.16). For other activities, participants’ choices did not differ from chance (Listening to music: 25/60, 41.67%, *p* = .245, OR = .71; Cooking: 23/60, 38.33%, *p* = .092, OR = .62; Exercising: 27/60, 45.0%, *p* = .519, OR = .82; Bonferroni-corrected alpha level = .0083; see Figure 8). There were no significant effects of character gender on intelligence choices (*F* = .112, *p* = .738, nested model comparison)

Participants also reliably selected the aesthetically-motivated character as more capable of experiencing physical sensations than functionally-motivated matched characters (233/360, 64.7%, *p* < .001, OR = 1.83, binomial test vs. chance proportion of 50%). At the individual activity level, after correcting for multiple comparisons, this effect only held when the activity was listening to music (41/60, 68.3%, *p* = .006, OR = 2.16; Bonferroni-corrected alpha level = .0083). Two other activities showed smaller effects that did not reach this conservative alpha threshold (Painting: 40/60, 66.7%, *p* = .013, OR = 2.00; Cooking: 39/60, 65.0%, *p* = .027, OR = 1.86; Exercising: 39/60, 65.0%, *p* = .027, OR = 1.86). The remaining activities did not differ from chance (Being in nature: 36/60, 60.0%, *p* = .155, OR = 1.50; Doing math: 38/60, 63.3%, *p* = .052, OR = 1.73; see Figure 8). Overall, there was no significant effect of activity (*F* = .217, *p* = .956) or character gender (*F* = 1.48, *p* = .224, nested model comparisons) on these choices.

#### Model Comparison: Intelligence, Not Emotionality, Predicts Helpfulness Judgments.

As predicted, judgments about mental capacities were significant predictors of participants’ helpfulness judgments. However, the predictors for helpfulness differed from those of compassionate behavior. When emotionality judgements were added as a predictor (to the null model predicting helpfulness judgments as a function of activity and stimulus character gender), they did not add significant predictive power (emotionality: *F* = 1.34, *p* = .247; nested model comparisons vs. a null model; see Table 3). In contrast, when added individually to the null model, both intelligence (*F* = 17.86, *p* < .001) and physical experience capacities (*F* = 4.60, *p* = .032) added significant predictive power.

**Table 3.** Model Comparison Results, Experiment 4

**Predictors of Helpful Behavior**	**AIC Value of Model (lower implies better fit)**	**Nested Model Comparisons vs. first model**	
		***F***	***p***
Activity, Gender	486.22	–	–
Intelligence, Activity, Gender	470.35	17.86	<.001
Physical Experience, Activity, Gender	483.61	4.60	0.032
Emotional Experience, Activity, Gender	486.88	1.34	0.247
Intelligence, Physical Experience, Activity, Gender	466.76	11.73	<.001
Emotional Experience, Intelligence, Activity, Gender	469.27	10.47	<.001
Emotional Experience, Physical Experience, Activity, Gender	485.35	2.43	0.088
Emotional Experience, Intelligence, Physical Experience, Activity, Gender	467.61	8.20	<.001

Using AIC as a measure of model fit, with lower AIC values indicating better-fitting models, intelligence judgments best predicted helpfulness judgments (*AIC* = 470.35), doing so better than the model including only emotionality (*AIC* = 486.88; *p* < .001, analysis of Akaike weights to test whether intelligence model has a significantly lower AIC) or only physical experience capacities (*AIC* = 483.61; *p* = .0013, analysis of Akaike weights to test whether intelligence model has a significantly lower AIC).

A model including both intelligence and physical experience capacities as predictors had the lowest raw AIC value (*AIC* = 466.76) compared to models containing any other combination of the three mental capacities. However this model was not significantly better than the simpler model containing only intelligence as a predictor (*p* = .166, analysis of Akaike weights to test whether the larger model has a significantly lower AIC).

#### Demand Effects Check: Participants Remained Unaware of Hypothesis.

When participants were asked what they thought the study was about, five of 360 mentioned the critical characters specifically, and four mentioned that we had manipulated characters’ hobbies or occupations (inter-rater reliability = 100%; using the same free response coding method as in previous experiments). All findings remain the same whether these participants’ data are included or excluded, with no differences in conclusions or statistical significance. As preregistered, results reported include these data points.

### Discussion

We find that aesthetic motivations do not reliably increase attributions of helpfulness, in spite of reliably increasing expectations of compassionate behavior. Thus, all prosocial traits may not be impacted in the same manner by observations about others’ aesthetic vs functional motivations. In particular, consistent with the idea that effective helping requires intelligence and skill, not just prosocial intentions, we find that helpfulness judgments were most strongly predicted by attributions of intelligence, and that emotionality and physical experience capacities did not provide any additional explanatory power. This contrasts with judgements of compassionate behavior, which were best predicted by the capacity for emotional experience. Overall, this supports the idea that observers’ behavioral predictions about aesthetically-motivated people are mediated by broader attributions of mental capacities (including intelligence, as well as the capacity for experience).

## GENERAL DISCUSSION

Overall, across four pre-registered, highly-powered experiments, we show that others’ aesthetic appreciation and aesthetic motivation conveys important social information about them. Compared to matched characters performing the same activity for a functional reason, people robustly and reliably judged aesthetically-motivated characters as more likely to behave compassionately (Exp. 1, 3), less likely to behave selfishly or manipulatively (Exp. 1, 3), having a greater sensitivity to emotion (Exp. 2, 3, 4) and (to a lesser extent) more capable of physical sensations (Exp. 2, 3, 4; with differences across activities). These attributions of increased prosociality and sensitivity held across a broad range of activities and contexts, including both conventional art forms (painting, music), and activities not considered conventional art forms (exercise, cooking, doing math, and being in nature). As hypothesized, others’ aesthetic motivation impacted interpersonal attributions above and beyond simply engaging in a particular behavior (like painting). Participants reliably judged characters as more prosocial, less antisocial, and more sensitive when the person had an atelic goal, finding intrinsic value in the activity itself, rather than valuing the activity as the means to an end (Pigliucci, 2019; Schachner & Carey, 2013; Schmid, 2011). In philosophy, this tendency to value things for their own sake has been theorized to predict an individual’s own moral aptitude (Jaworska, 1999; Watson, 2013), and empirical work has supported this idea that people who engage in aesthetic activities behave more prosocially (e.g., Dollinger & LaMartina, 1998; Kou et al., 2020; Van de Vyver & Abrams, 2018). Here we find that this atelic or aesthetic motivation impacts fundamental interpersonal attributions, as well.

There are marked individual differences in the extent to which people engage in aesthetic activities, and in the extent to which people’s behaviors are motivated by aesthetic appreciation (Fayn et al., 2015; Schlotz et al., 2021). Our findings suggest that these real-world individual differences in aesthetic motivations impact attributions of fundamental components of mental life (experience, intelligence) and inferences about others’ prosociality. This likely includes negative judgements about individuals who are rarely or never aesthetically motivated. For example, in previous work focusing solely on musicality, non-musical individuals were judged less emotionally sensitive, and less wrong to harm, than neutral baseline characters or musical individuals (Agrawal et al., 2023). Thus, the impact of aesthetic motivation on attributions of mental capacities may have broad consequences for social interaction.

Our causal model helps explain why and how aesthetic motivations are linked with attributions of increased prosociality. In particular, we find evidence that attributions of increased emotional sensitivity best predict judgments of compassionate behavior (Exp. 3). These findings provide support for the idea that broad judgments about mental capacities, particularly regarding increased emotional sensitivity, mediate the impact of aesthetic motivation on judgements of prosocial behavior. This may be a rational inference: Choosing to spend time or money on an activity for no other reason than finding it intrinsically beautiful or enjoyable may provide a signal that the person is motivated by emotional experience, and may therefore have greater emotionality in general. In contrast, choosing to engage in an activity for a functional reason does not provide evidence that the person experiences strong emotions, as there is an alternative explanation for why they are engaging in the activity (i.e., to achieve the desired functional outcome). Overall, these findings provide empirical support for the idea that lay people have sentimentalist intuitions mirroring the views of 18th century philosophers (Heinzelmann et al., 2020; Hume, 1910): We seem to intuitively believe that emotionality is a key driver of both aesthetic and moral behavior.

Prior work has explored prosocial effects of engaging in various artistic activities (Konrath & Kisida, 2021), including their potential to prevent conflict and promote peace in context of large-scale societal conflicts (e.g., Clarke et al., 2015). One previously unrecognized but important causal factor may be *why* people engage in those artistic activities. Individuals who are aesthetically-motivated are likely to be regarded as more compassionate and less selfish, as compared to those engaged in the same artistic activities for externally-motivated reasons (e.g., to earn a paycheck; to gain earlier release from prison). The current work lays the foundation for future studies to explore how our effects extend to real-world interpersonal contexts, particularly whether arts interventions have stronger social and interpersonal effects when individuals engage in them for aesthetic vs. functional reasons. These findings may help in designing more effective, scalable interventions for increasing prosocial behavior in real-world contexts.

Our experiments included a broad range of participants in terms of age and location across the U.S., and several internal replications, suggesting that these findings are likely robust and generalizable within this cultural context. Future work should explore how far these effects extend across cross-cultural and economically-diverse populations, and the extent to which these inferences vary. Cultures around the world appreciate aesthetic beauty (Che et al., 2018; Schellekens & Goldie, 2011; Tooby & Cosmides, 2001). However, the extent to which people value functionality varies cross-culturally (e.g., Lim & Ang, 2008), and the Kantian conception of aesthetic appreciation as “disinterested” (i.e., free from external goals) has usually been applied in the context of elite, “leisure-class” Western societies (Sharman, 1997). Certain non-Western cultures, such as the Yolngu bark-painters of Australia (Morphy, 1989) or the Ghanaian Asante carvers (Silver, 1979), value functionality over form even in the context of aesthetic objects, and do not judge aesthetic objects as being ‘beautiful’ or ‘ugly’ in and of themselves (Sharman, 1997). Future work with people from cultures that place similar versus different value on aesthetic vs. functional goals should probe the extent to which the interpersonal inferences studied here generalize across cultures, and the extent to which these inferences differ outside of the U.S. cultural context.

### Judgments About Intelligence, and the Relation to Helpfulness

In contrast to judgments of compassionate behavior, participants did not select the characters with aesthetic motivation as more intelligent. Instead, they either did not choose systematically between the aesthetically- and functionally-motivated characters, or reliably chose aesthetically-motivated characters as *less* intelligent than functionally-motivated ones (Exp. 2, 3, and 4). We believe that the functionally-motivated characters were likely viewed as having higher than typical intelligence, rather than viewing aesthetically-motivated individuals as lacking normal intelligence. This would make sense: By definition, the functionally- motivated characters were described as motivated by practical goals. Having goals in place and acting to achieve them provides clear evidence of intelligence, by showing that the individual is able to plan, make decisions, and produce intentional, controlled behaviors (Duncan et al., 1996; Hommel, 2017).

We see interesting differences in intelligence judgments across different populations tested. University undergraduates (at a top public university in Southern California with a high proportion of Asian and Hispanic students, and strength in science and research) reliably chose aesthetically-motivated characters as less intelligent than matched functionally-motivated characters (Exp. 2, 4). In contrast, when our sample involved a broader demographic of participants with respect to age, ethnicity, and geographic location, participants did not reliably choose either of our matched characters as more intelligent than the other (Exp. 3). One possibility is that undergraduate students at highly-rated U.S. universities may value practical, functional goals and achievements more so than the broader public.

The dissociation between intelligence judgements and aesthetic motivation provided a test case for us to explore whether aesthetic motivation leads to increased attribution of all prosocial behaviors, and to provide an additional test of whether observers’ judgements about aesthetically-motivated people are mediated by broader attributions of mental capacities (including intelligence, as well as emotional sensitivity). We found that aesthetic motivations did not lead to an expectation of increased helpfulness, in spite of an expectation for more compassionate behavior (Exp. 4). This finding was predicted by two factors: 1) Unlike compassionate behavior, helpful behavior requires not only motivation to help, but also the intelligence to help effectively (Dovidio, 1984); and 2) our finding that aesthetically-motivated people are not perceived as more intelligent than functionally-motivated individuals (Exp. 2, 3, 4). In line with this, participants’ intelligence judgments best predicted their attributions of helpfulness—in contrast to their attributions of compassionate behavior, which were better predicted by attributions of emotional sensitivity. These data provide convergent evidence for the idea that broad judgments of mental capacities mediate the impact of aesthetic motivation on judgements of social behavior.

These data also point out the importance of considering affective versus cognitive components of prosocial behavior. Prosocial behavior consists of distinct socio-affective and socio-cognitive components (Preckel et al., 2018). Socio-affective components refer to emotional traits like empathy and compassion; while socio-cognitive components include mental processes like perspective-taking, theory of mind, and other broader abilities to intelligently reason about people’s beliefs, goals, and mental states. Behaving prosocially may require one or both of these capacities, depending on the context and type of prosocial behavior (Imuta et al., 2022; Preckel et al., 2018; Schurz et al., 2021).

This framework helps explain the differing results of Exp. 3 and 4, and enables us to clarify the scope of our effects. Compassion is a predominantly affective trait (because it requires an individual to experience emotions in response to observing others in distress; Stellar et al., 2015), and compassionate behavior may primarily be explained by this affective component. We find that individuals who appreciate aesthetic beauty are judged as being more emotionally sensitive. They thus may be judged more likely to experience prosocial feelings, like compassion and empathy, towards others; this may motivate judgements that they would behave in a compassionate manner, as well. In contrast, helpfulness may require an additional cognitive component, as the ability to be helpful towards others requires the intelligence and skill to help effectively (Dovidio, 1984; Imuta et al., 2016). This framework provides a foundation for future work to assess the extent to which aesthetic vs. functional motivations impact inferences about other prosocial traits and behaviors, like empathy, fairness, and generosity (Cuff et al., 2014; McAuliffe et al., 2017; Pfattheicher et al., 2022).

## ACKNOWLEDGMENTS

We thank Pascal Gagneux, Joshua Rottman, and Michael McCullough for helpful feedback on this project. This work was supported by funding from the Grammy Foundation’s Research Grant to AS, as well as a fellowship to TA from the Center for Academic Research and Training in Anthropogeny (CARTA) at the University of California, San Diego.

## Note


^1 ^
This similarity question was inspired by the experimental design in Agrawal et al. (2023), who used it to check if participants simply chose characters due to similarity attraction (which was found not to be the case). In the current studies, participants chose whether they were more similar to a character doing an activity for aesthetic vs. functional reasons. Since most people have both kinds of motivations in their daily lives, we determined that this measure was unlikely to be informative, and do not analyze or report these data here.
